# Supplementary material for: Targeting TGFβR2‐mutant tumors exposes vulnerabilities to stromal TGFβ blockade in pancreatic cancer
Source: EMBO Mol Med. 2019 Oct 14;11(11):e10515. doi: 10.15252/emmm.201910515 (PMC6835203; doi:10.15252/emmm.201910515)

Appendix

Table of content

Appendix Table S1. Gene list of mouse RT^2 Profiler PCR Array

Appendix Supplementary Figure Legends

Appendix Figure S1. Mouse chemokine changes in human xenografts with stromal TGFβR2 blockade.

Appendix Figure S2. Chemokine changes in *KIC* tumors with TGFβR2 blockade.

Appendix Figure S3. TGFβ promotes LIF secretion from fibroblasts and the expression of fibroblast markers in human CAFs.

Appendix Figure S4. Effect of TGFβ and IL-6 on EMT in cancer cells.

Appendix Figure S5. Validation of NK cell depletion effect of anti-Asialo-GM1 antibody.

Appendix Figure S6. Therapeutic efficacy of TGFβR2 or IL-6 inhibition in xenografts is NK cell dependent.

Appendix Figure S7. Cytotoxic T-cell spatial distribution analysis.

Appendix Figure S8. Graphic synopsis: TGFβ-IL-6 paracrine signal from CAFs stimulates cancer cells and inhibit NK cell function in TGFβR2-mutant PDA.

Appendix Table S1. Gene list of mouse RT^2 Profiler PCR Array

Symbol	Description
Ackr3	Chemokine (C-X-C motif) receptor 7
Aicda	Activation-induced cytidine deaminase
Bcl2	B-cell leukemia/lymphoma 2
Bcl2l1	Bcl2-like 1
Ccl2	Chemokine (C-C motif) ligand 2
Ccl20	Chemokine (C-C motif) ligand 20
Ccl22	Chemokine (C-C motif) ligand 22
Ccl28	Chemokine (C-C motif) ligand 28
Ccl4	Chemokine (C-C motif) ligand 4
Ccl5	Chemokine (C-C motif) ligand 5
Ccr1	Chemokine (C-C motif) receptor 1
Ccr10	Chemokine (C-C motif) receptor 10
Ccr2	Chemokine (C-C motif) receptor 2
Ccr4	Chemokine (C-C motif) receptor 4
Ccr5	Chemokine (C-C motif) receptor 5
Ccr7	Chemokine (C-C motif) receptor 7
Ccr9	Chemokine (C-C motif) receptor 9
Cd274	CD274 antigen
Csf1	Colony stimulating factor 1 (macrophage)
Csf2	Colony stimulating factor 2 (granulocyte-macrophage)
Csf3	Colony stimulating factor 3 (granulocyte)
Ctla4	Cytotoxic T-lymphocyte-associated protein 4
Cxcl1	Chemokine (C-X-C motif) ligand 1
Cxcl10	Chemokine (C-X-C motif) ligand 10
Cxcl11	Chemokine (C-X-C motif) ligand 11
Cxcl12	Chemokine (C-X-C motif) ligand 12
Cxcl2	Chemokine (C-X-C motif) ligand 2
Cxcl5	Chemokine (C-X-C motif) ligand 5
Cxcl9	Chemokine (C-X-C motif) ligand 9
Cxcr1	Chemokine (C-X-C motif) receptor 1
Cxcr2	Chemokine (C-X-C motif) receptor 2
Cxcr3	Chemokine (C-X-C motif) receptor 3
Cxcr4	Chemokine (C-X-C motif) receptor 4
Cxcr5	Chemokine (C-X-C motif) receptor 5
Egf	Epidermal growth factor
Egfr	Epidermal growth factor receptor
Fasl	Fas ligand (TNF superfamily, member 6)
Foxp3	Forkhead box P3
Gbp2b	Guanylate binding protein 1

Gzma	Granzyme A
Gzmb	Granzyme B
H2-D1	Histocompatibility 2, D region locus 1
H2-K1	Histocompatibility 2, K1, K region
Hif1a	Hypoxia inducible factor 1, alpha subunit
Ido1	Indoleamine 2,3-dioxygenase 1
Ifng	Interferon gamma
Igf1	Insulin-like growth factor 1
Il10	Interleukin 10
Il12a	Interleukin 12A
Il12b	Interleukin 12B
Il13	Interleukin 13
Il15	Interleukin 15
Il17a	Interleukin 17A
Il1a	Interleukin 1 alpha
Il1b	Interleukin 1 beta
Il1r1	Interleukin 1 receptor, type I
Il2	Interleukin 2
Il22	Interleukin 22
Il23a	Interleukin 23, alpha subunit p19
Il4	Interleukin 4
Il5	Interleukin 5
Il6	Interleukin 6
Irf1	Interferon regulatory factor 1
Kitl	Kit ligand
Mif	Macrophage migration inhibitory factor
Myc	Myelocytomatosis oncogene
Myd88	Myeloid differentiation primary response gene 88
Nfkb1	Nuclear factor of kappa light polypeptide gene enhancer in B-cells 1, p105
Nos2	Nitric oxide synthase 2, inducible
Pdcd1	Programmed cell death 1
Ptgs2	Prostaglandin-endoperoxide synthase 2
Spp1	Secreted phosphoprotein 1
Stat1	Signal transducer and activator of transcription 1
Stat3	Signal transducer and activator of transcription 3
Tgfb1	Transforming growth factor, beta 1
Tlr2	Toll-like receptor 2
Tlr3	Toll-like receptor 3
Tlr4	Toll-like receptor 4
Tlr7	Toll-like receptor 7
Tlr9	Toll-like receptor 9

Tnf	Tumor necrosis factor
Tnfsf10	Tumor necrosis factor (ligand) superfamily, member 10
Trp53	Transformation related protein 53
Vegfa	Vascular endothelial growth factor A

Appendix Figure S1. Mouse chemokine changes in human xenografts with stromal TGFβR2 blockade. ELISA-based Quansys mouse chemokine assay was performed with Capan-1 (Cap), MiaPaCa-2 (Mia), Colo357 (Colo), and C5LM2 (C5) orthotopic tumor samples treated with saline (control) or 2G8 (n = 3/group).

Appendix Figure S2. Chemokine changes in *KIC* tumors with TGFβR2 blockade. *KIC* mice were treated for 4 weeks with Mac84 (control) or 2G8. Tumors were collected for ELISA-based Milliplex mouse chemokine screening (n = 2/group).

Appendix Figure S3. TGFβ promotes LIF secretion from fibroblasts and the expression of fibroblast markers in human CAFs. (A) NIH 3T3 cells were treated with TGFβ (30 ng/ml) or TGFβ + 2G8 (100 ng/ml) for 24 hours. Conditioned media was collected and subjected to mouse LIF ELISA. P value by ANOVA is shown. **(B)** Cell lysates from human CAF cell lines CAF-PC1 and CAF-PC2 were harvested and levels of αSMA, PDGFRα and tubulin determined by western blotting.

Appendix Figure S4. Effect of TGFβ and IL-6 on EMT in cancer cells. *KIC* (mPLRB9) and *KPC* (KPC-M09) cell lines were treated with IL-6 (100 ng/ml), TGFβ (30 ng/ml), IL-6 plus TGFβ for 24 hours and 48 hours, respectively. The level of EMT markers E-cadherin, N-cadherin, vimentin, and actin was determined by western blotting.

Appendix Figure S5. Validation of NK cell depletion effect of anti-Asialo-GM1 antibody. C57BL/6 mice received 50 μg of control rabbit IgG or anti-Asialo-GM1 three days in a row. Splenocytes were harvested and subjected to flow cytometry for the detection of CD335 positive NK cells. n=5/group, P value by t test.

Appendix Figure S6. Therapeutic efficacy of TGFβR2 or IL-6 inhibition in xenografts is NK cell dependent. Human pancreatic cancer cell line Colo357 was orthotopically implanted into NOD SCID or NSG mice. After tumor establishment, mice were randomized to receive Rat IgG Mac84 (control), 2G8 or anti-mouse IL-6 antibody MP5-20F3 (each 30 mg/kg, 2x/week, n = 7-10/group) for 3.5 weeks. Tumors were harvested for analysis and gross metastases were counted. Total gross metastases were determined by evaluation of liver at the time of sacrifice. P values by t test is shown.

Appendix Figure S7. Cytotoxic T-cell spatial distribution analysis. Formalin-fixed, paraffin-embedded tumor tissue slides were stained for CD8. A machine learning algorithm was used to isolate the IHC stain from the counterstain. Then a tissue segmentation algorithm was used to exclude the glass background, followed by nucleus detection and cell simulation algorithms to segment and detect the IHC-positive and negative cells. IHC-positive cells were then classified into low, medium, and high, based on the signal intensity.

Appendix Figure S8. TGFβ-IL-6 paracrine signal from CAFs stimulates cancer cells and inhibit NK cell function in TGFβR2-mutant PDA.

Appendix Figure S1

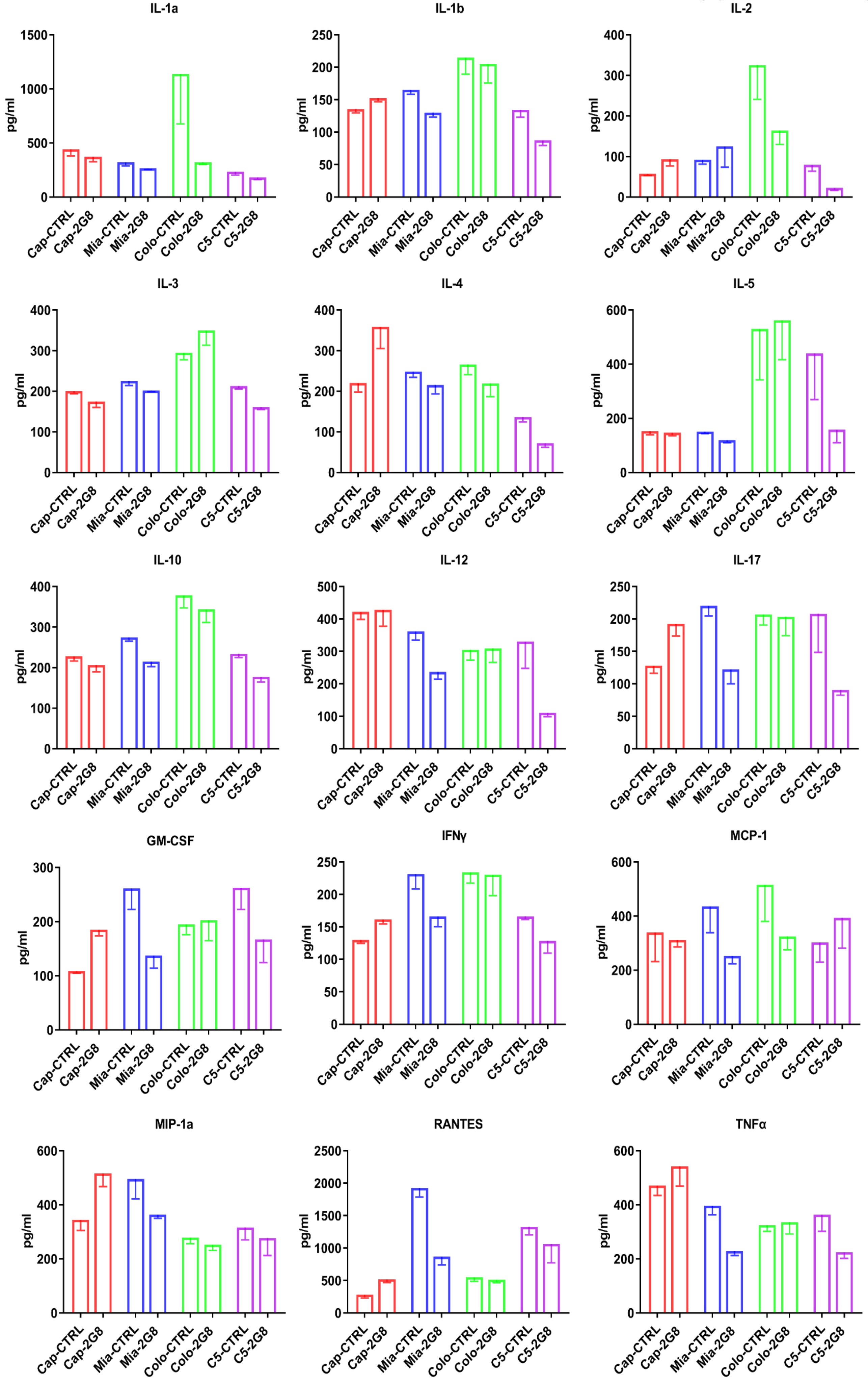

Appendix Figure S2

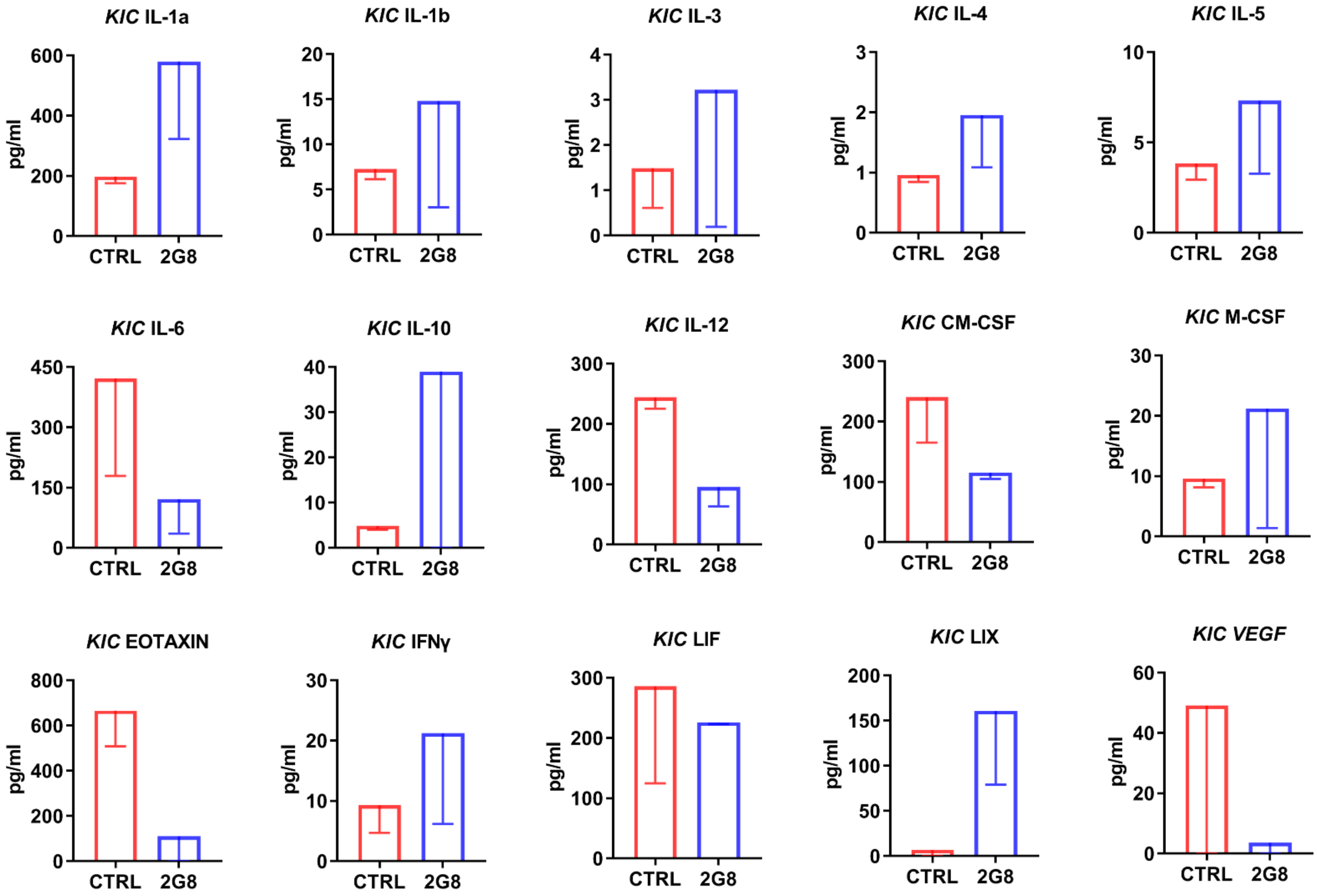

Appendix Figure S3

A

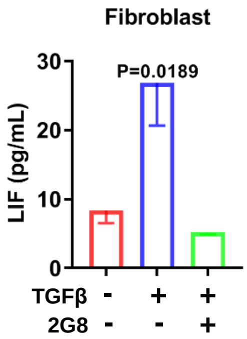

B

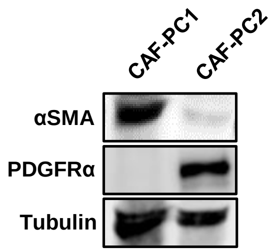

Appendix Figure S4

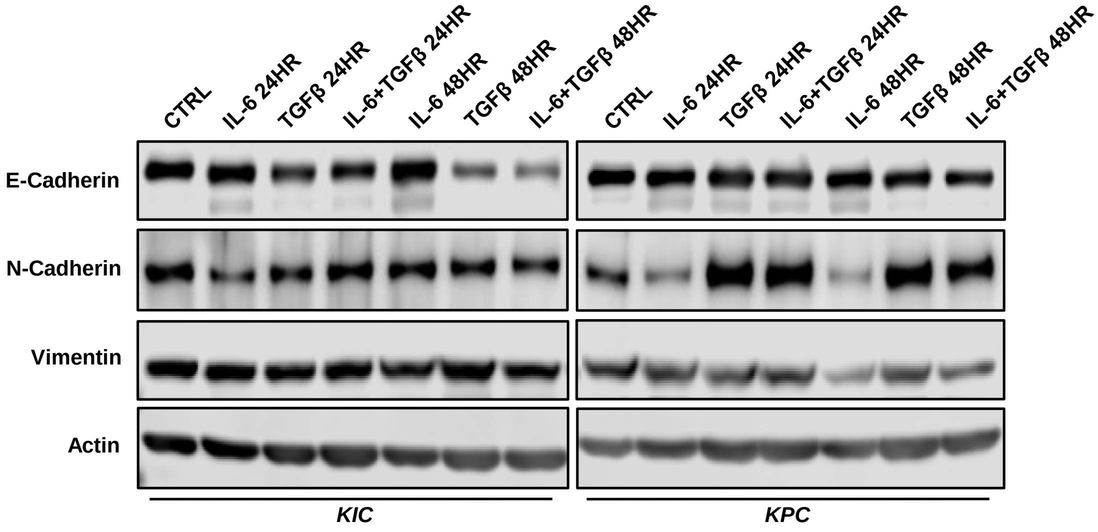

Appendix Figure S5

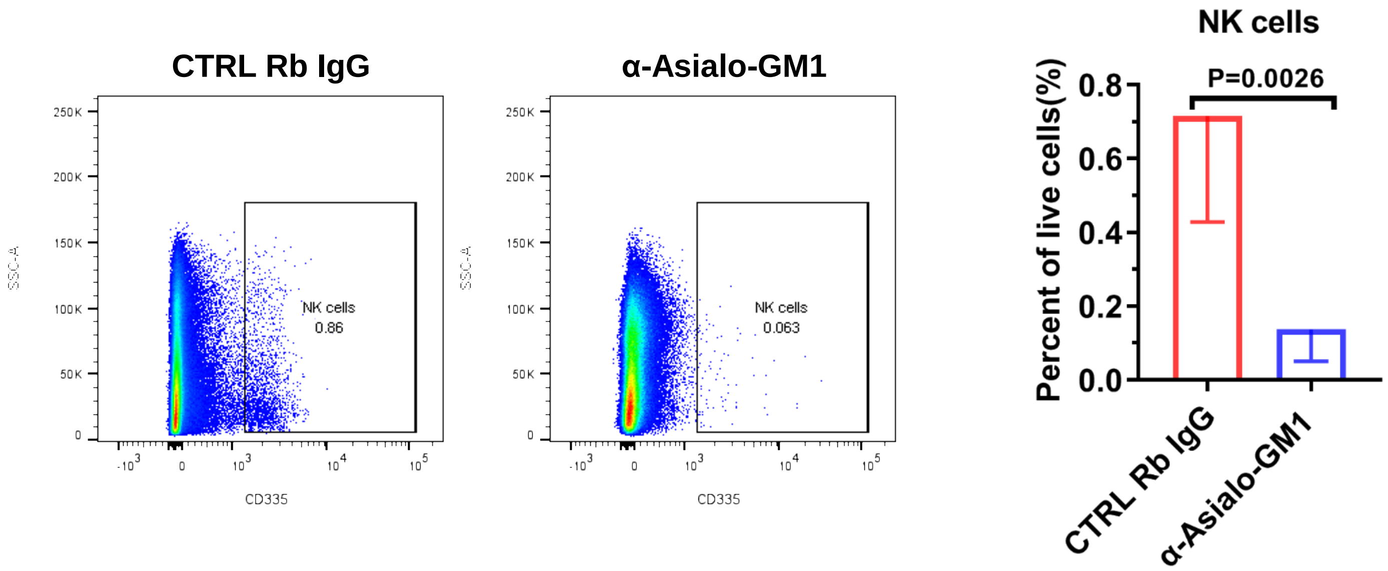

Appendix Figure S6

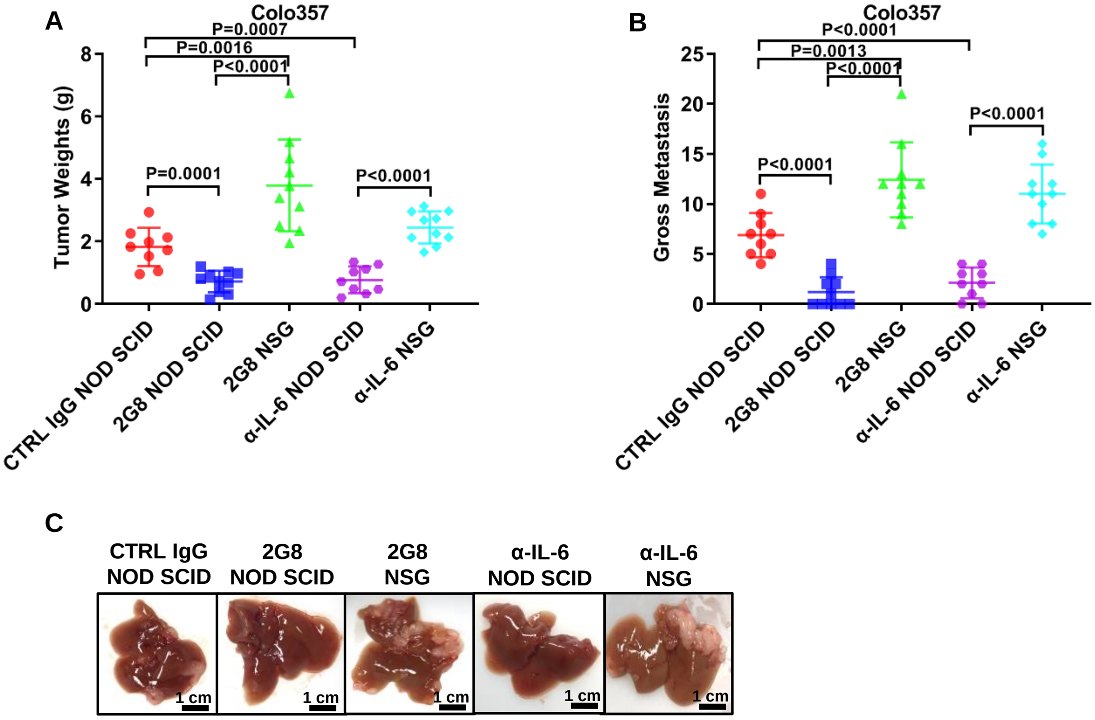

Appendix Figure S7

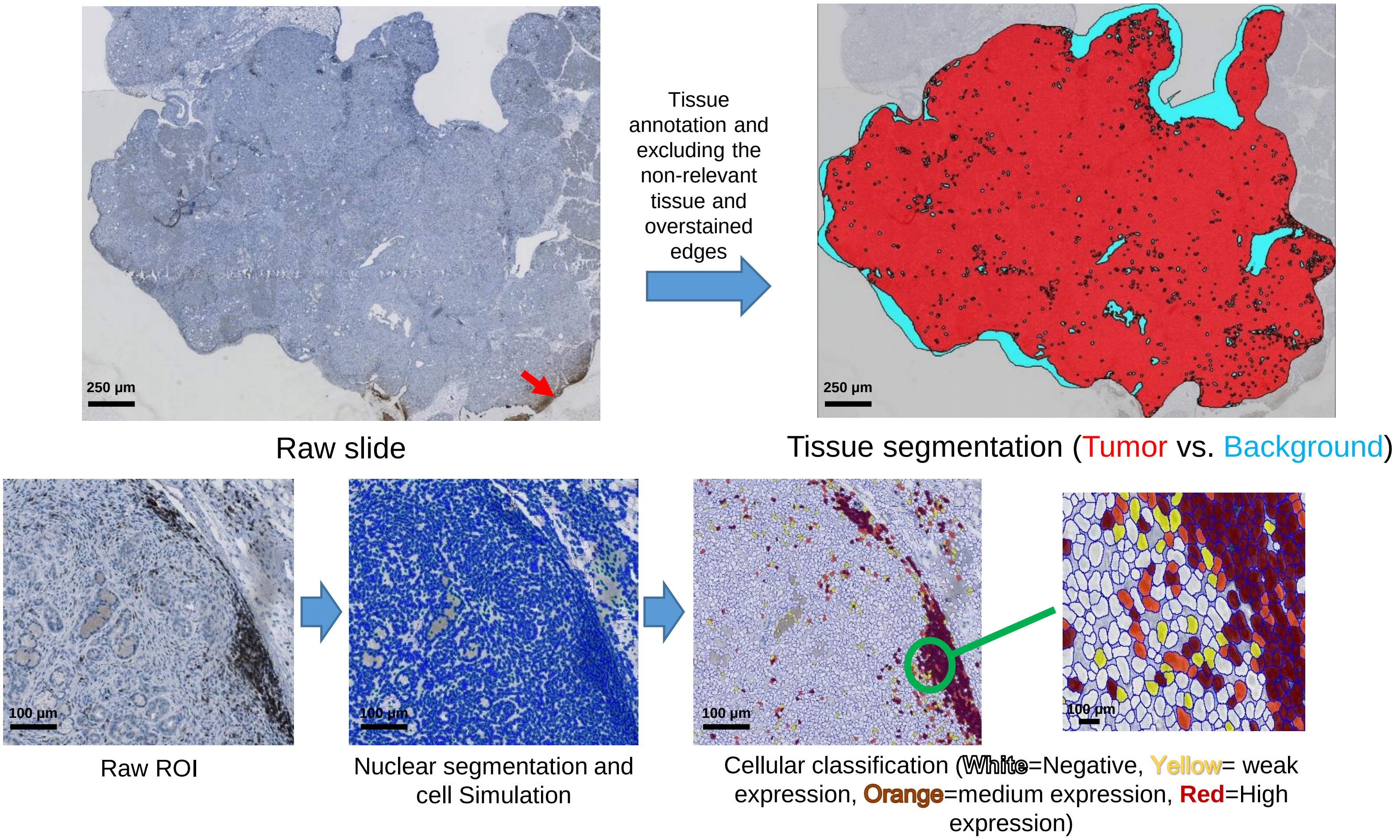

Appendix Figure S8

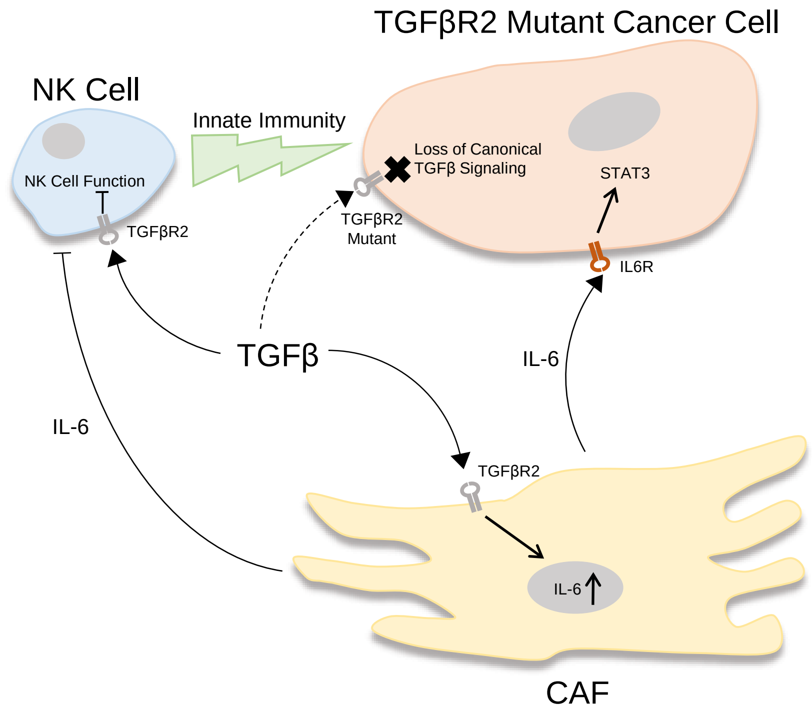

Supplement: Supplementary file 1 — Appendix [file EMMM-11-e10515-s001.pdf]
