# Supplementary figures and images for: Targeting TGFβR2‐mutant tumors exposes vulnerabilities to stromal TGFβ blockade in pancreatic cancer
Source: EMBO Mol Med. 2019 Oct 14;11(11):e10515. doi: 10.15252/emmm.201910515 (PMC6835203; doi:10.15252/emmm.201910515)

Appendix Figure S3B

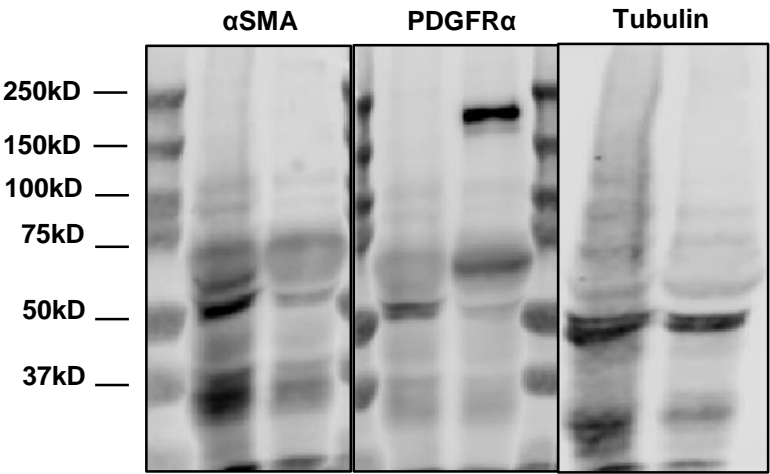

Supplement: Supplementary file 2 — Source Data for Appendix [file EMMM-11-e10515-s006.zip › Appendix_source_data/FigS3_source_data.pdf]

Source data for Appendix Figure S4

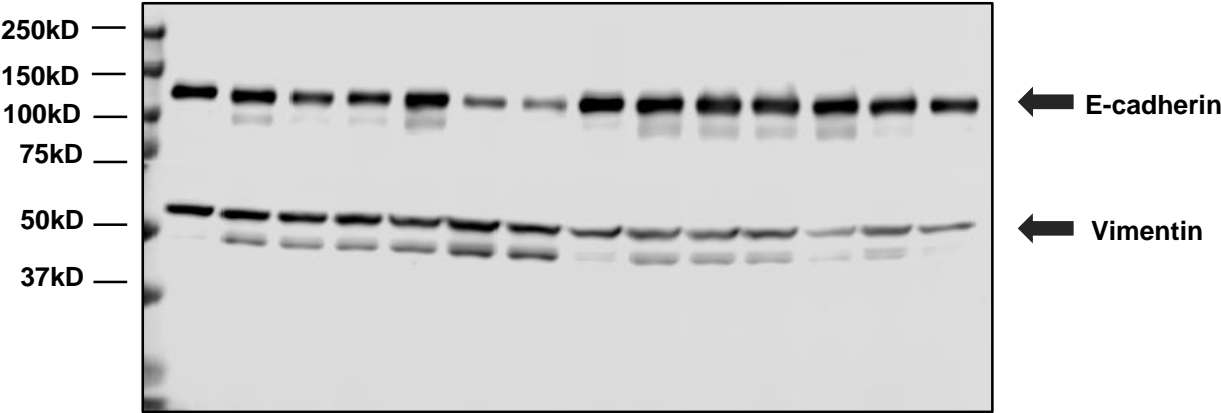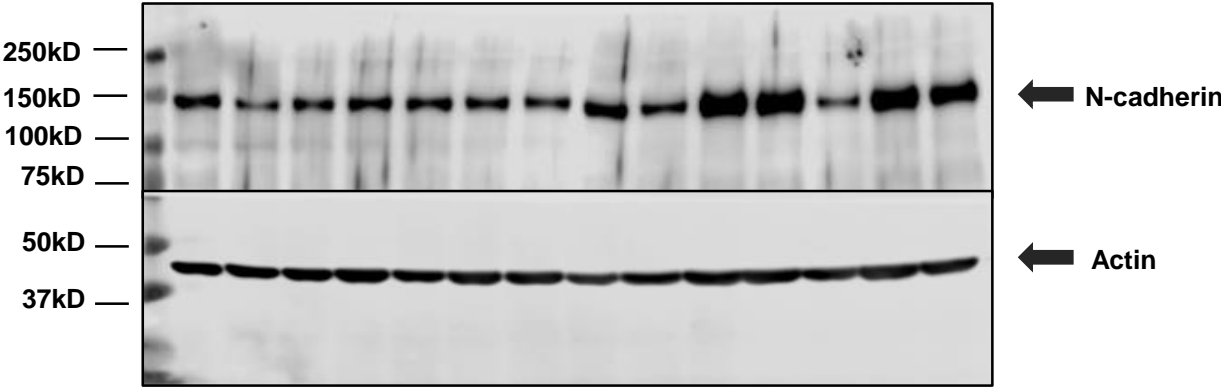

Supplement: Supplementary file 2 — Source Data for Appendix [file EMMM-11-e10515-s006.zip › Appendix_source_data/FigS4_source_data.pdf]

Source data for Figure 2

Figure 2B

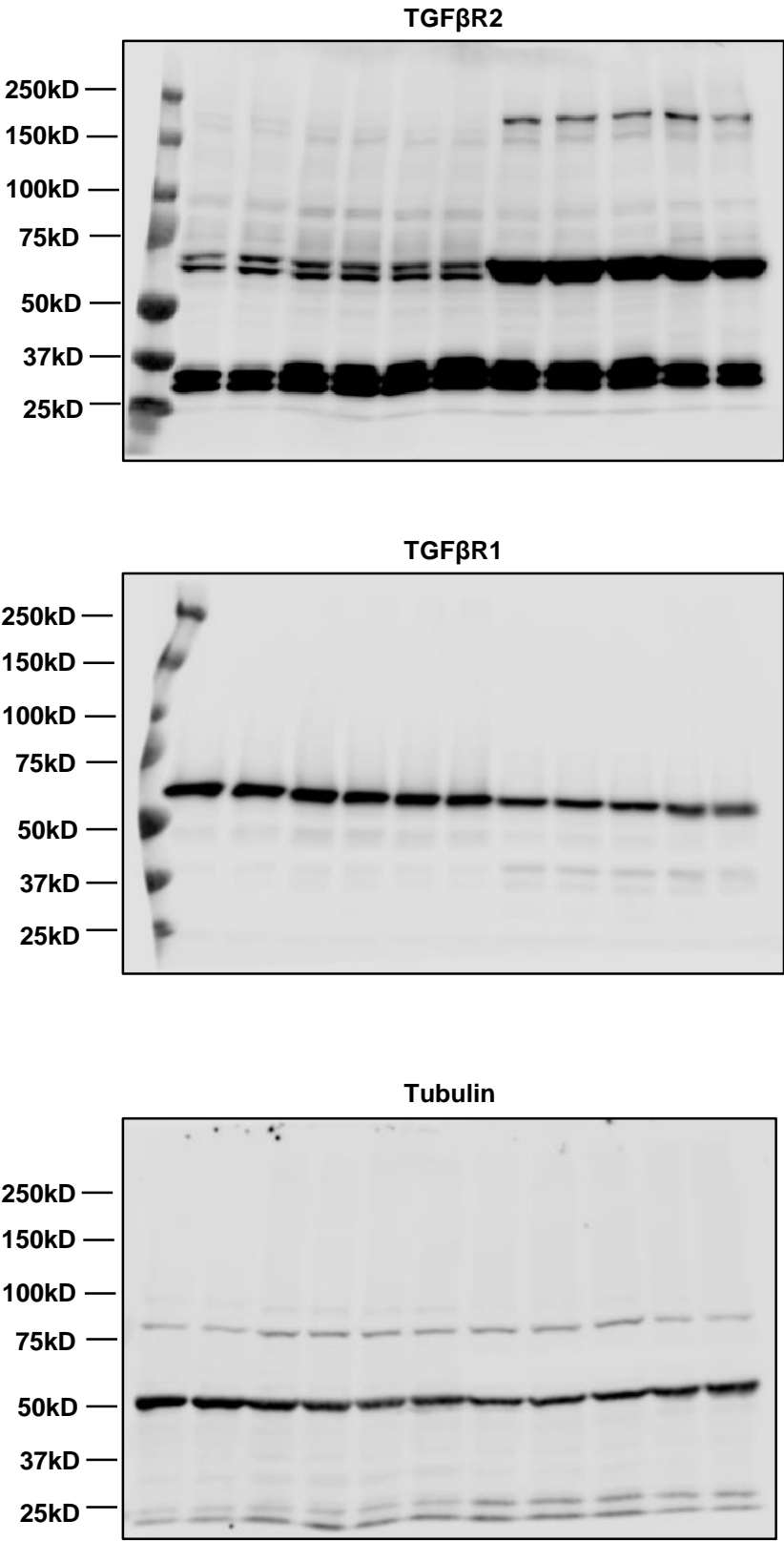

Supplement: Supplementary file 4 — Source Data for Figure 2 [file EMMM-11-e10515-s002.pdf]

Source data for Figure 3

Figure 3A

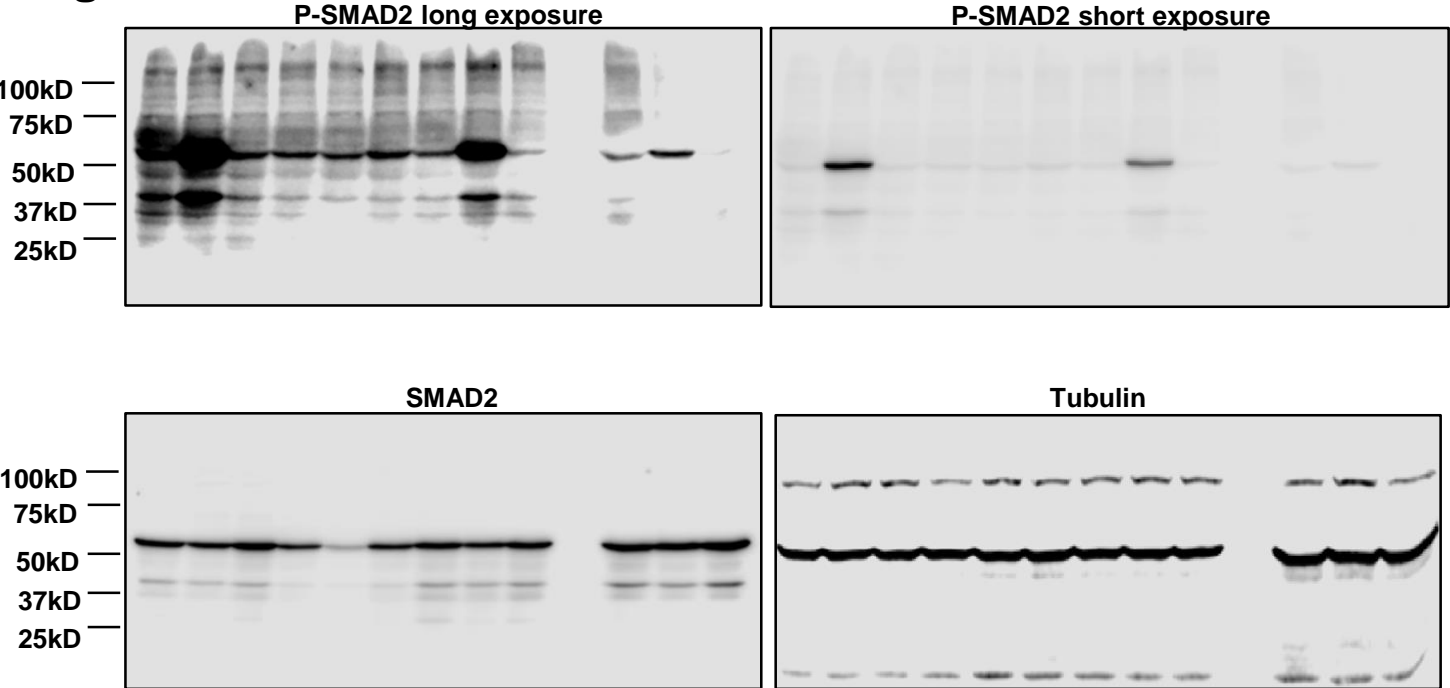

Figure 3J

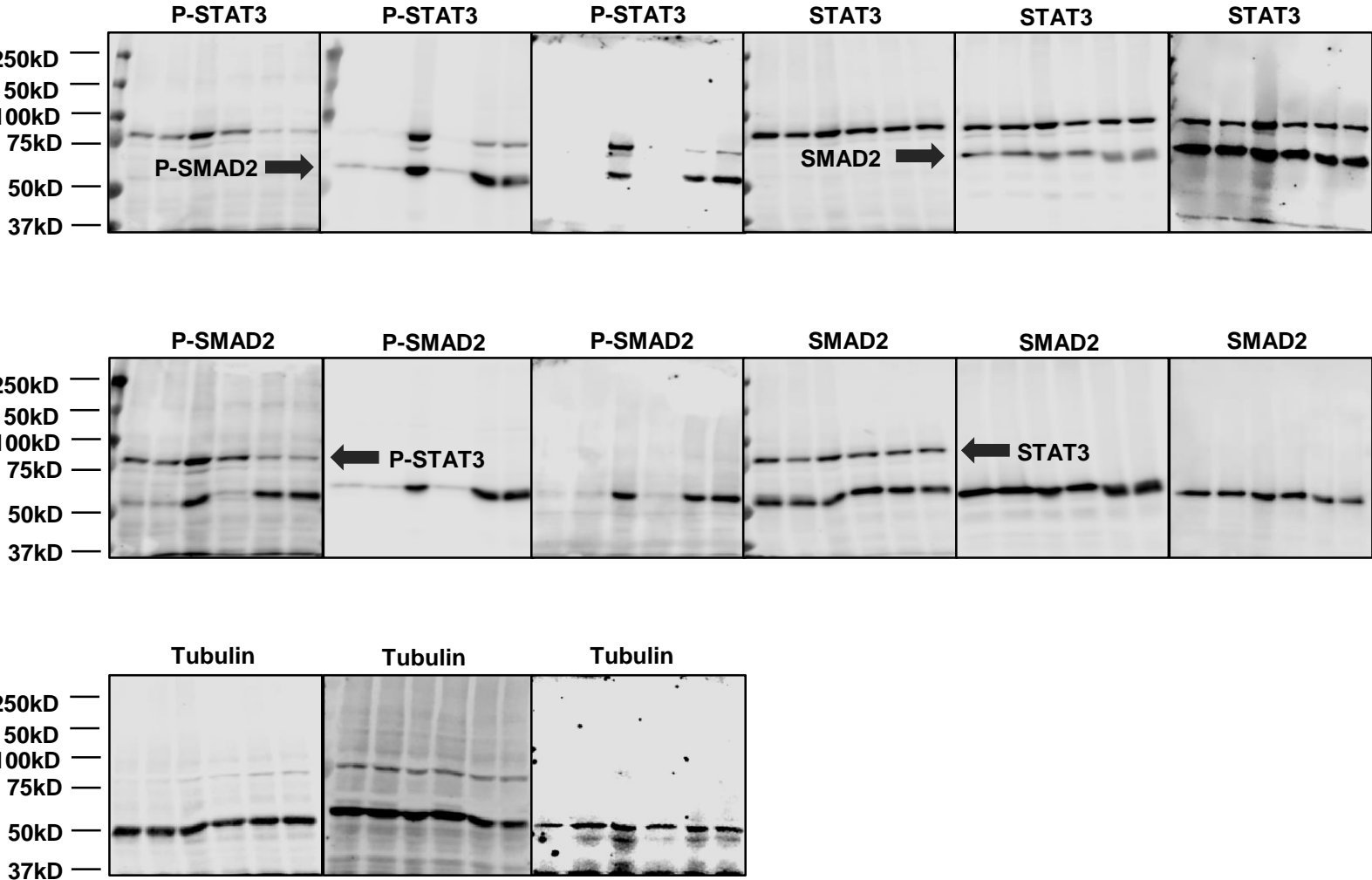

Supplement: Supplementary file 5 — Source Data for Figure 3 [file EMMM-11-e10515-s003.pdf]

Source data for Figure 6

Figure 6F

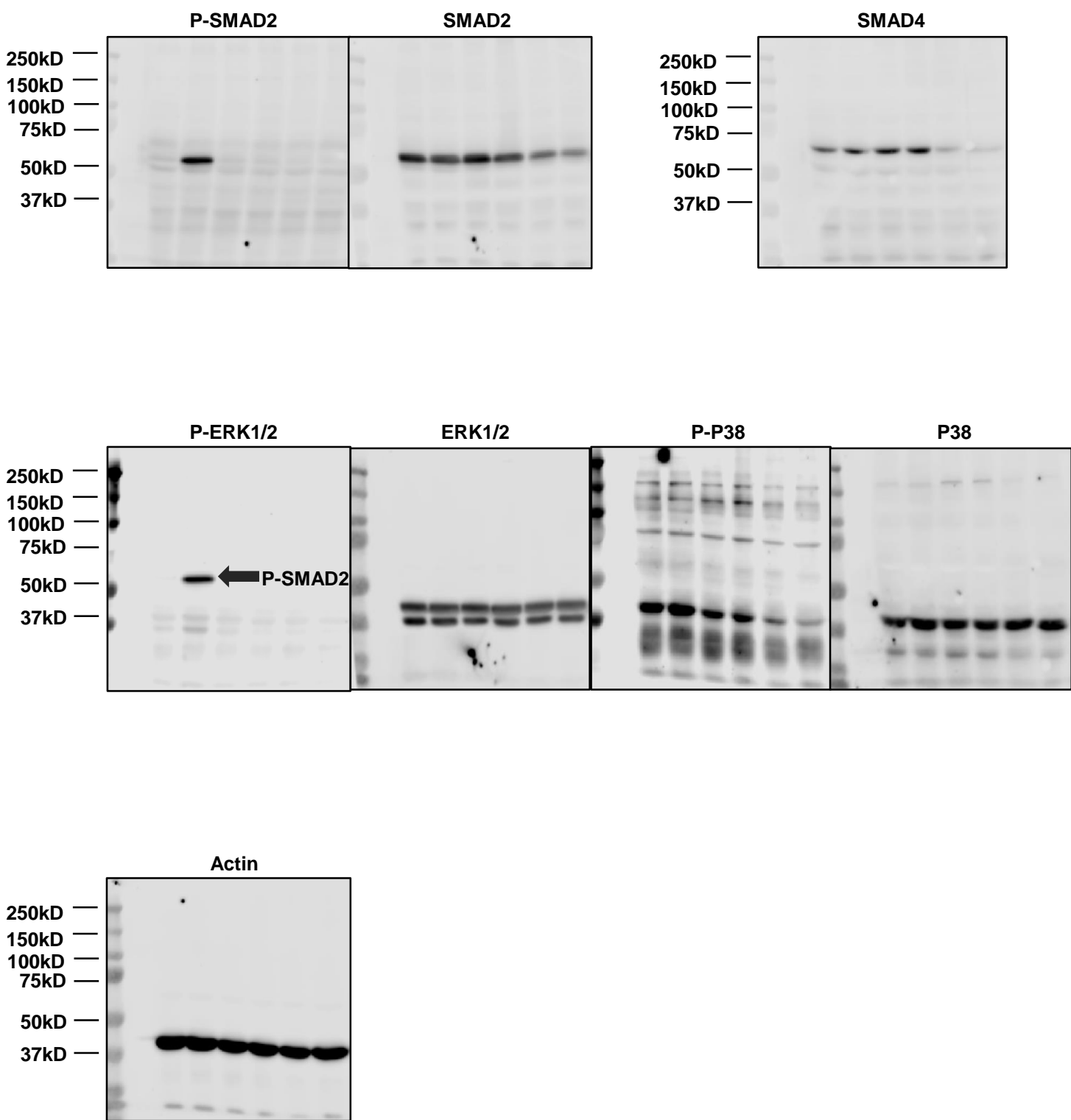

Supplement: Supplementary file 6 — Source Data for Figure 6 [file EMMM-11-e10515-s004.pdf]

Source data for Figure 8

Figure 8A

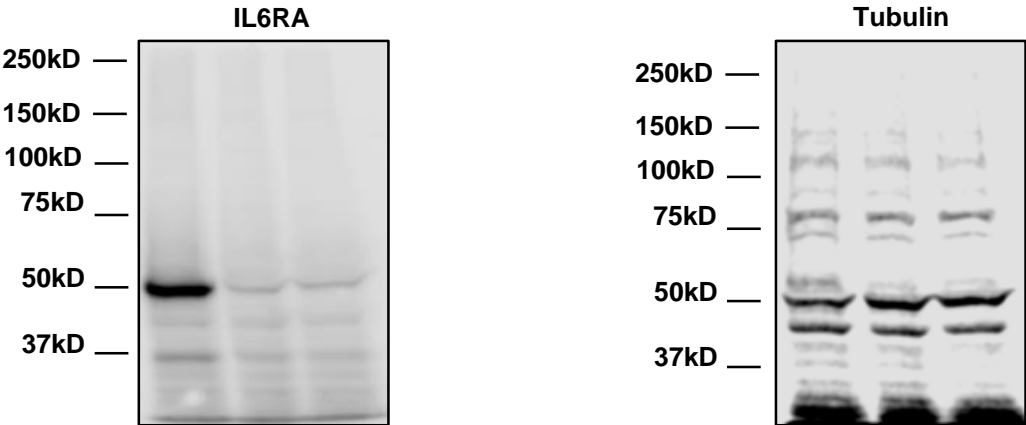

Supplement: Supplementary file 7 — Source Data for Figure 8 [file EMMM-11-e10515-s005.pdf]
